# Distance between the supraspinatus and long head of the biceps tendon on sagittal MRI: a new tool to identify anterior supraspinatus insertion injury

**DOI:** 10.1186/s40634-021-00410-6

**Published:** 2021-11-25

**Authors:** Lifeng Yin, Hua Zhang, Yangang Kong, Xinyu Zhang, Wenlong Yan, Jian Zhang

**Affiliations:** Department of Orthopaedics, The First Affiliated Hospital of Chongqing Medical University, NO.1 Youyi Road, Chongqing, 400016 Yuzhong District China

**Keywords:** Rotator cuff tear, Magnetic resonance imaging, Supraspinatus central tendon, Long head of the bicep tendons, Anterior supraspinatus insertion

## Abstract

**Purpose:**

Anterior insertion of the supraspinatus muscle plays an essential role in rotator cuff tissue. We aimed to determine whether the distance between the midpoints of the supraspinatus central tendon and long head of the biceps tendon on a sagittal shoulder magnetic resonance imaging scan can help to preoperatively diagnose an injury of the anterior insertion of the supraspinatus.

**Method:**

This retrospective study reviewed 103 patients with a full-thickness supraspinatus tendon tear: 50 patients with (injured group) and 53 patients without (intact group) anterior supraspinatus insertion tear. The inter-tendon distance was measured based on an oblique sagittal magnetic resonance imaging scan. SPSS was used for statistical analyses. Two independent samples t-test and receiver operating curve analysis were also performed.

**Results:**

The measurements of inter-tendon distance revealed good intra- and inter-observer reliabilities with intra-class correlation coefficients of 0.92 and 0.97, respectively. The inter-tendon distance of the injured group was significantly greater than that of the intact group (10.1 ± 2.7 vs 8.0 ± 2.3 mm, *P* < 0.001). The diagnostic ability of the inter-tendon distance was fair (area under the curve = 0.745), and a threshold of 9 mm had a specificity of 73% and sensitivity of 74%.

**Conclusion:**

The distance between the supraspinatus central tendon and long head of the biceps tendon on magnetic resonance imaging was greater in patients with anterior supraspinatus insertion injury than those without the injury. A distance of 9 mm may be the cut-off value and a good diagnosis marker for anterior supraspinatus insertion injury.

**Level of evidence:**

Level III, diagnostic case–control study.

## Introduction

Rotator cuff tear, usually associated with shoulder pain and dysfunction, is a common shoulder condition [16]. Injuries of the supraspinatus tendon can be assessed by magnetic resonance imaging (MRI) clearly [4, 9]. For patients with full-thickness injuries, shoulder arthroscopy is a good treatment option.

Recent studies have reported that the anterior insertion of the supraspinatus muscle plays an essential role in supraspinatus function [2, 3, 6, 8, 10, 12, 14]. Currently, the area anterior to the greater tuberosity and near the intertubercular sulcus is considered as the anterior insertion point of the supraspinatus muscle [10, 13]. Biomechanical studies have demonstrated that injury to the anterior supraspinatus muscle significantly affects stress distribution [6, 10, 11]. Further, clinical studies observed that anterior supraspinatus tears are often accompanied by more severe tears, muscle atrophy, and re-tearing after repair [2, 3]. Therefore, complete preoperative understanding of the anterior supraspinatus tear is critical for preoperative diagnosis and intraoperative repair strategies.

MRI scan is a standard method for the preoperative evaluation of tendon and muscle injuries, especially rotator cuff tears. Previous studies have reported an association of the posterior displacement of the supraspinatus central tendon with an injury to the insertion point of the anterior supraspinatus [19]. However, the use of an image overlap to observe the position of the central tendon poses some difficulties in clinical procedures.

Clinically, we observed that the distance between the supraspinatus central tendon and long head of the biceps tendon (LHBT) often varies; the tendons are sometimes close or further apart. We consider this may be related to anterior supraspinatus insertion injury.

The purpose of this study was to propose a novel indirect parameter imaging method to assist in the preoperative diagnosis of anterior supraspinatus insertion injury. We hypothesized that the central distance between the LHBT and supraspinatus tendon on an oblique sagittal MRI was correlated with an injury of the anterior supraspinatus tendon and could be used to assist preoperative diagnosis of anterior supraspinatus injuries.

## Materials and method

### Study patients

This retrospective study was approved by the institutional review board (NO. 2021–07). Participants were divided into the injury group and intact group according to intraoperative observations. A 5-mm area close to the intertubercular groove of the great tubercle was considered the anterior insertion of the supraspinatus [10, 15].

The inclusion criteria were as follows: (1) patients who had undergone a primary rotator cuff repair at our institution between 2015 and 2020, (2) had clear MRI data of the shoulder and documented tear pattern and size in their operative report, (3) no history of shoulder surgery or trauma, (4) full-thickness supraspinatus or infraspinatus tendon tear, and (5) front to back size of tear of 1–3 cm.

The exclusion criteria were as follows: (1) advanced glenohumeral joint arthritis, (2) tendon retracted over the top of the humeral head, (3) extreme internal and external rotation of the humeral head on a shoulder MRI scan [19], (4) dislocation or rupture of the LHBT, (5) intraoperative subscapularis tendon repair, and (6) frozen shoulder.

### Shoulder MRI scan

A 1.5- or 3.0-T Siemens MRI scanner (Siemens, Erlangen, Germany) was used examine the shoulder. The MRI scan of the patient’s shoulder included an axial turbo spin-echo (TSE) proton density-weighted image and TSE T1- and T2-weighted images with fat suppression on a parallel or sagittal oblique plane to the supraspinatus tendon.

### Measurement on MRI

All measurements were performed by two independent orthopedic resident surgeons. One musculoskeletal radiologist provided help, and one senior orthopedic doctor supervised. All measurements were performed on the picture archiving and communication system of our hospital (IDX Image cast, version 10.7; Koninklijke Philips Electronics, Amsterdam, Netherlands). The same surgeon repeated the measurements 6 weeks after the initial measurements to assess intra-observer reliability.

**Fig. 1:**
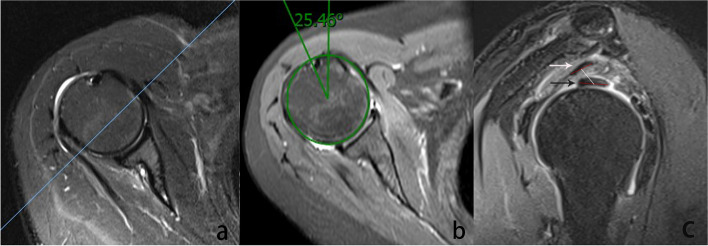
**a** First, an oblique sagittal cross-section across the tip of coracoid process was selected (blue line). **b** On a horizontal plane, a proximal slice depicting the obvious outline of the bicipital groove was selected to measure the radius and rotation angle (green line). The angle formed by the line from the central point to the bicipital groove and the vertical line was defined as rotation angle. **c** The white arrow indicates the supraspinatus central tendon, and the black arrow indicates the long head of the biceps tendon. From the anterior edge to the posterior edge of each tendon two straight lines (red line) are drawn. Inter-tendon distance was defined as distance (white line) between the midpoints of the two straight lines

Fat-suppressed T2 or proton-density fat-suppressed sagittal sequence of the MRI scan was reviewed, and inter-tendon distance was measured as the distance between center of the supraspinatus central tendon and LHBT on the oblique sagittal MRI, where the coracoid process begins to disappear (Fig. 1 a). The location of the supraspinatus central tendon and LHBT can be identified by continuously observing oblique sagittal images [20]. A straight line connecting the anterior edge and the posterior edge of each tendon was drawn respectively. The distance between midpoints of the two straight lines was recorded as inter-tendon distance (Fig. 1 c).

On a horizontal plane, a proximal slice depicting an obvious outline of the bicipital groove was chosen to measure the radius and to locate the central point of the humeral head that was then used to measure the rotation angle. The angle formed by the line from the central point of the humeral head to the bicipital groove and the vertical line was defined as the rotation angle. It was stipulated that the internal rotation was positive, and the external rotation was negative [19] (Fig. 1 b).

### Tear characteristic and fatty muscle degeneration

The tear size was categorized into two degrees: level 1 (1–2 cm) and level 2 (2–3 cm). Supraspinatus muscle atrophy was ranked using the Goutallier grading criteria [7]: Level 0, no fat infiltration; Level 1, few fat bands on the muscles; Level 2, less fat than muscle; Level 3, fat equivalent to the muscle; and Level 4, more fat than the muscle.

### Sample size and statistical methods

Since there was no similar previous study to determine the variables, we performed a post-hoc sample size power analysis using Power and Sample Size 11 (329 North 1000 East Kaysville, Utach 84,037 USA). The results revealed that the sample size of our study was sufficient with a power of > 99% and effect size > 0.8 (*P* < 0.05).

Mean values and standard deviations were used to express continuous data, and frequencies and percentages were used to express categorical data. Two-tendon distance and other parameters were compared between the groups using the independent t-test. Classification and rank variables were compared using the χ^2^ test. Correlation analysis, inter- and intra-observer consistency analysis, and receiver operating curve analysis were also performed.

Statistical significance was set at a *P*-value of < 0.05.

## Results

Overall, 103 patients were included in this study: 50 with injury (age 60.4 ± 8.6 years, 22 males and 28 females) and 53 without injury (age 55.3 ± 10.8 years, 25 males and 28 females). No significant difference was observed in the baseline data between the two groups. Table 1 summarizes the baseline information and measurement results of the two groups.

**Table 1:** Demographic information and magnetic resonance imaging measurement results

Variables	Anterior intact	Anterior injured	*P*-value
Number	53	50	NA
Age (years)	55.3 ± 10.8	60.4 ± 8.6	> 0.05
Sex			
Male	25 (47%)	22 (44%)	> 0.05
Female	28 (53%)	28 (56%)	
BMI (kg/m^2^)	23.0 ± 3.5	24.2 ± 3.7	> 0.05
Tear size			
1–2 cm	25 (47%)	23 (46%)	> 0.05
2–3 cm	28 (53%)	27 (54%)	
Muscle atrophy 0/I/II/III	**15/35/3/0**	**5/24/20/1**	**< 0.01**
Inter-tendon distance (mm)	**8.0 ± 2.3**	**10.1 ± 2.7**	**< 0.01**
Humeral head radius (mm)	21.9 ± 1.8	21.5 ± 1.8	> 0.05
Rotation angle (°)	5.0 ± 18.5	10.1 ± 15.5	> 0.05

*Bold values indicate statistical significance (*P* < 0.05)

*MRI* Magnetic resonance imaging, *BMI* Body mass index

**Fig. 2:**
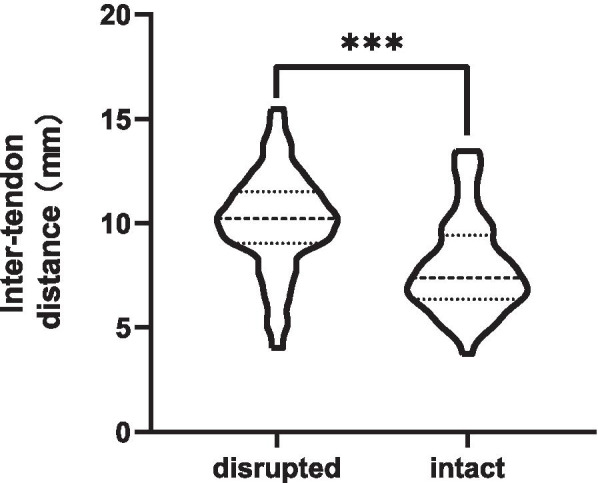
Differences in inter-tendon distance between the two groups. Inter-tendon distance in patients with anterior supraspinatus muscle injury was significantly larger than that in the intact group

The inter-tendon distance significantly increased in the injury group (10.1 ± 2.7 mm) compared with that in the intact group (8.0 ± 2.3 mm, *P* < 0.01, Fig. 2). Meanwhile, the degree of supraspinatus muscle atrophy was more severe in the injury group (*P* < 0.05) than in the intact group.

**Fig. 3:**
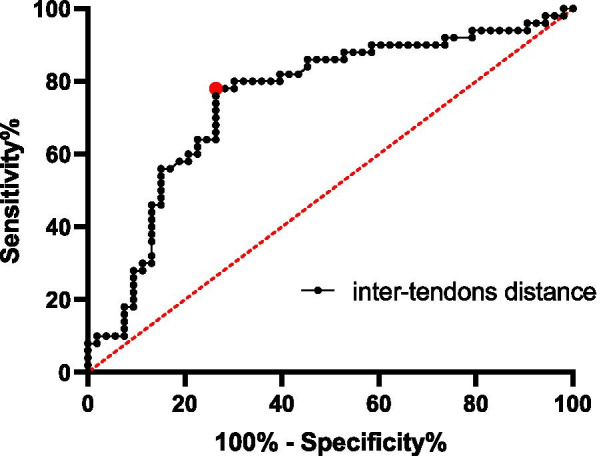
Receiver operating curve and calculation of the area under the curve also indicate that inter-tendon distance has a good ability in predicting anterior supraspinatus insertion injury (AUC = 0.746)

Receiver operating curve analysis indicated that the distance could help in the diagnosis of anterior insertion injury (AUC = 0.746). When 9 mm was selected as the cut-off value, 74% sensitivity and 73% specificity were obtained (Fig. 3). This distance also showed good inter-observer and intra-observer consistencies (after 6 weeks) with intra-class correlation coefficients of 0.92 and 0.97 (*P* < 0.05), respectively.

## Discussion

The key finding of this study was a novel imaging parameter that may be used to assist in the assessment of anterior supraspinatus insertion injury preoperatively. On the oblique sagittal shoulder MRI scan, the area where the coracoid process just disappears, the central distance between the supraspinatus central tendon and LHBT was greater when the tears involved the anterior area than when it involved other areas, and it depicted a good ability to diagnose anterior injury when a 9-mm distance was set as the cut-off value.

Previous studies have reported that anterior supraspinatus insertion plays an essential role in shoulder biomechanics [2, 3, 6, 10, 12, 14]. This study indicated that patients with damaged anterior insertion were more likely to develop more severe symptoms of supraspinatus muscle atrophy and re-tear after a surgical repair than those that by patients without damage [1, 2]. Therefore, it is mandatory to properly understand the insertion of the supraspinatus preoperatively.

Gray et al. observed that the supraspinatus central tendon is constantly oriented toward the bicipital groove in normal shoulders, and it was usually posteriorly displaced when the tear affected the insertion of the anterior supraspinatus tendon [19]. However, their method required a selection of the MRI slice with an appropriate overlap. In this study, because the assessment parameters were simple, they were evaluated using a single MRI slice.

Variation in the two-tendon distance may be due to the central tendon insertion injury, which is located in the area of the anterior supraspinatus [17, 19]. This may explain the significant difference in the two-tendon distance and supraspinatus muscle atrophy severity observed between the two groups. Most studies have overlooked the supraspinatus central tendon. A recent study reported detachment of the supraspinatus central tendon from its insertion to be responsible for a functional deficit of the upper limb and the central tendon plays a vital role in arm abduction by transmitting force through an intact rotator cuff muscle [17]; hence, reinsertion of the central tendon in the correct anatomical location is desirable to optimize functional outcome after surgery [18].

This study has some limitations. First, we excluded patients with an internal or external rotation of angel > 30° and with a tear size of > 3 cm or a tendon retracted over the humeral head. Second, the MRI slice thickness of 4 mm may have influenced the plane of measurement chosen for patients. The lateral and interior plane may increase and reduce the distance between tendons, respectively. More importantly, the MRI images in this study were retrospectively analyzed; therefore, the distal plane of the coracoid process, which is the standard plane of measurement, could not be accurately identified. More accurate detection methods are needed to confirm the results of this study. Third, anatomic variations of the coracoid process has been reported among different populations. An anatomic study found that the length and base width of the coracoid process was 42.47 ± 1.02 mm and 23.90 ± 0.76 mm, respectively, in the Chinese population, which is different among other Asian populations [5], These observed variations may influence the generalizability of the results of this study; thus, the findings of this study may not be applicable in other populations. Further research is warranted to determine whether inter-tendon distance correlates with central tendon injury.

## Conclusion

The distance between the supraspinatus central tendon and LHBT on MRI is associated with anterior supraspinatus insertion injury, and a cut-off value of 9 mm may inform the diagnosis of this injury.

## Data Availability

The datasets used and the analysis during the current study are available from the corresponding author on reasonable request.
